# Identification of conserved and novel microRNAs in *Porphyridium purpureum* via deep sequencing and bioinformatics

**DOI:** 10.1186/s12864-016-2985-7

**Published:** 2016-08-11

**Authors:** Fan Gao, Fangru Nan, Jia Feng, Junping Lv, Qi Liu, Shulian Xie

**Affiliations:** School of Life Science, Shanxi University, Taiyuan, 030006 China

**Keywords:** miRNA, RNA-seq, sRNA, *Porphyridium purpureum*

## Abstract

**Background:**

*Porphyridium purpureum* has been utilized in important industrial and pharmaceutical fields. The identification of microRNAs (miRNAs) in this unique species is of great importance: such identification can help fill gaps in the small RNA (sRNA) studies of this organism and help to elucidate essential biological processes and their regulation mechanisms in this special micro alga.

**Results:**

In this study, 254 high-confidence miRNAs (203 conserved miRNAs and 51 novel miRNAs) were identified by sRNA deep sequencing (sRNA-seq) combined with bioinformatics. A total of 235 putative miRNA families were predicted, including 192 conserved families and 43 species-specific families. The conservation and diversity of predicted miRNA families were analysed in different plant species. Both the 100 % northern blot validation rate (VR) of four randomly selected miRNAs and the results of stem-loop quantitative real time RT-PCR (qRT-PCR) assays of 25 randomly selected miRNAs demonstrated that the majority of the miRNAs identified in this study are credible. A total of 14,958 and 2184 genes were predicted to be targeted by the 186 conserved and 41 novel miRNAs. Gene ontology (GO) annotation and Kyoto Encyclopedia of Genes and Genomes (KEGG) pathway analysis indicated that some target genes likely provide valuable references for further understanding of vital functions in *P. purpureum.* In addition, a cytoscape network will provide some clues for research into the complex biological processes that occur in this unique alga.

**Conclusions:**

We first identified a large set of conserved and novel miRNAs in *P. purpureum*. The characteristic and validation analysis on miRNAs demonstrated authenticity of identification data. Functional annotation of target genes and metabolic pathways they involved in illuminated the direction for further utilization and development this micro alga based on its unique properties.

**Electronic supplementary material:**

The online version of this article (doi:10.1186/s12864-016-2985-7) contains supplementary material, which is available to authorized users.

## Background

*Porphyridium purpureum* (Bory) Drew et Ross is the a special unicellular micro-red alga with strong tolerance for variations in salinity, growing in seawater, saltwater, freshwater or wetlands [1]. *P. purpureum* is the same species as *P. cruentum* (Gray) Nag., first discovered by Nasegli in 1849 [2]. Although it is an age-old microalga*, P. purpureum* has received much attention since the 1980s as a result of its unique contributions to the chemical and pharmaceutical industries [3]. *P. purpureum* may represent a potential source of industrial raw materials, especially bioactive substances such as polysaccharides [4], B-phycoerythrin [5] and polyunsaturated fatty acid (PUFA) [6].

Previous studies have confirmed that exo-polysaccharides (EPS) from *Porphyridium* can inhibit various viruses in vitro and *vivo*, including A/So-fia/80/92/H3N2 [7], herpes simplex virus (HSV)-1 and HSV-2 [8], varicella zoster virus (VZV) [9], respiratory syncytial virus (RSV) [10], and coxsackie virus B3 (CVB3) [11]). It has also been demonstrated that EPS from *Porphyridium* can significantly improve mouse immunity by enhancing spleen cell proliferation and triggering natural killer cell (NK) and interleukin-2 (IL-2) cell activity [12]. Researchers also observed that EPS is approximately 6.55 kDa in mass, contains more than 40 % of the sulfate in *Porphyridium*, and plays a distinct role in enhancing immunocompetence in vitro. In addition, EPS from *Porphyridium* exhibits anti-tumour effects: the 6.55 and 256 kDa EPS have exhibited inhibitory effects against lung adenocarcinoma cells (A549), laryngeal human epidermoid cancer cells (Hep-2) and human hepatoma cells spontaneous monocyte-mediated cytotoxity 7721 (SMMC7721) [13].

As a natural biotic pigment, B-phycoerythrin in *Porphyridium* has been used as a food colorant and fluorescent protein marker in the food processing and biological medicine fields, respectively [14]. Some reports suggest that polyunsaturated fatty (PUFA) extracted from *Porphyridium* can be used as a substitute for deep sea fish oil, based on the ability of PUFA to lower body blood fat and cholesterol [15].

Although *P. purpureum* is a valuable alga resource, most studies to date have focused on species culturing [16], ecological function [17], pharmaceutical and food processing [18], phylogenetic evolution [19], and genome sequence analysis [20]. In recent years, botanical researchers have paid more and more attention to gene regulation at the post-transcriptional level [21]. We speculated that the biological and metabolic processes that produce these bioactive components might be regulated post-transcriptionally by non-coding sRNAs as well. However, no study on sRNAs in *P. purpureum* has been reported, and this lack of information will hinder the development and utilization of this unique species. Hence, it is necessary to carry out these sRNA studies in *P. purpureum.*

sRNA has been found to play a significant regulatory role in a wide range of organisms [22]. miRNAs, a well-studied subset of sRNAs, were first discovered in *Caenorhabditis elegans* in 1993 [23]. miRNAs are a class of small, non-coding, endogenous RNAs approximately 21 nt in length. miRNAs can negatively regulate gene expression at the post-transcriptional level by facilitating mRNA cleavage or by inhibiting translation to an extent based on the complementarity between the miRNA and its target [24]. Recent work has shown that differences exist between plant and animal miRNAs; these differences include the size of the miRNA and the precursor, the base bias of the miRNA, the position of the miRNA in the genome, and the processing and regulation methods of the miRNA [25]. Following the recent development of deep sequencing and bioinformatics, a large number of miRNAs have been identified in various plant species [26–32]. In addition, many studies have demonstrated that plant miRNAs fulfil essential functions in many biological processes, including growth and development [33], signal transduction [34], production of biological components [35], metabolism and response to stress [36]. Although a large number of miRNAs and their functions have been confirmed in model and higher plants, to date, nothing has been reported regarding miRNAs in *P. purpureum*.

To date, 38,499 miRNAs in 224 species have been identified and deposited in the miRNA database (miRBase 21.0, November 2015, http://www.mirbase.org/) [37]. Next-generation high-throughput sequencing combined with diverse bioinformatics methods may be an effective strategy for identifying as many *P. purpureum* miRNAs as possible based on the rich plant miRNA data contained in miRBase as well as the valuable genome sequences and rich expressed sequence tags (ESTs) of *P. purpureum* in GenBank [38]. Especially, the exclusive reference genome of *P. purpureum* (biosample ID: SAMN02981527) with about 19.45 M in length and 3014 contigs at assembly level has been published in 2014 [20]. Prediction of potential miRNA targets will be necessary for comprehensive mining of interesting target genes expressed during critical biological processes. In addition, information regarding miRNAs will facilitate the elucidation of some important biotic regulatory mechanisms and lay a foundation for further development and utilization of this special red alga [39].

## Results

### Characterization of sRNAs in *P. purpureum*

**Fig. 1:**
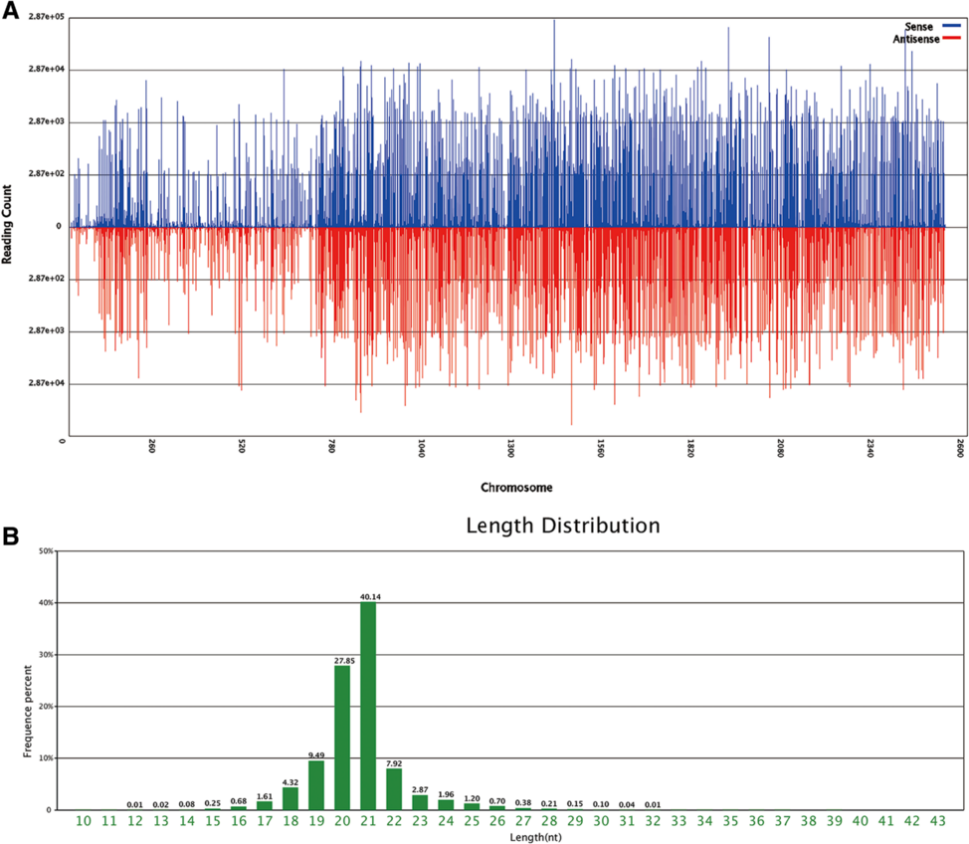
Positional and size distributions of sRNAs in *P. purpureum.* **a** Positional distribution of RNA-seq reads on the *P. purpureum* chromosome. It’s just a relative position sketch as a result of no any chromosome information about this alga having been published until now. Abundant of reads were distributed between contig780 and contig2500 as the figure shown. **b** Size distribution of sRNAs in *P. purpureum*. It’s a length distribution of RNA-seq reads in the sRNA library. As shown by their frequencies, 21-nt length reads were the most abundant

An sRNA library was constructed using RNA isolated from *P. purpureum* cultured in a photo bioreactor. This sRNA library was subjected to deep sequencing by Illumina HiSeqTM 4000, yielding a total of 11,425,306 raw reads. After filtering out contaminant and low-quality reads, 10,911,139 (95.76 %) clean reads remained (Table 1). Most of the sRNA tags were distributed between the 780 and 2500 contig region across the putative *P. purpureum* chromosomes (Fig. 1a). The lengths of the sRNAs varied widely from 10 to 43 nt. However, the majority of the sRNA reads were distributed between 19 and 22 nt (Fig. 1b). A length of 21 nt, the canonical length of plant miRNAs [40], was the most frequent size in the sRNA population (40.14 %). The numbers and proportions of unique and total small RNAs mapped to the *P. purpureum* genome are shown in Table 2. Approximately half (49.86 %) of the total reads and approximately one-fourth (25.79 %) of the unique reads were mapped. The average number of reads per unique sequence was 6.6. After removing rRNAs, tRNAs, snoRNAs, snRNAs and miRNAs based on matches to corresponding databases, only unannotated RNAs remained. The categories of sRNA are shown in Additional file 1: Figure S1A and B. The highest proportion of sRNAs were unannotated RNAs, with 9,949,268 redundant reads (1,560,880 unique reads). These unannotated RNAs were collected for further prediction of novel miRNAs. The raw reads could be queried by the numbers of SRX1631643 and SRR3228731 deposited in the NCBI Sequence Read Archive database.

**Table 1:** Summary of reads in small RNA libraries in *P. purpureum*

Type	Reads	Percent (%)
Total reads	11425306	
High quality	11394052	100 %
3’adapter null	754	0.01 %
Insert null	3337	0.03 %
5’adapter contaminants	179642	1.58 %
Smaller than 18 nt	298272	2.62 %
PolyA	908	0.01 %
Clean reads	10911139	95.76 %

**Table 2:** Summary of small RNAs mapped to genome in *P. purpureum*

	Unique reads	Percent (%)	Clean reads	Percent (%)
Total small RNAs	1646610	100 %	10911139	100 %
Mapping to genome	424606	25.79 %	5440437	49.86 %

### Conserved and novel miRNAs in *P. purpureum*

According to the aforementioned sRNA categories, matches were obtained for 327,434 redundant and 17,493 unique miRNA reads, accounting for 3 and 1.06 % of the redundant and unique miRNA reads, respectively. A total of 194 different known or conserved miRNAs were identified with an average of 86 unique reads per miRNA (Additional file 2: Table S1).

**Fig. 2:**
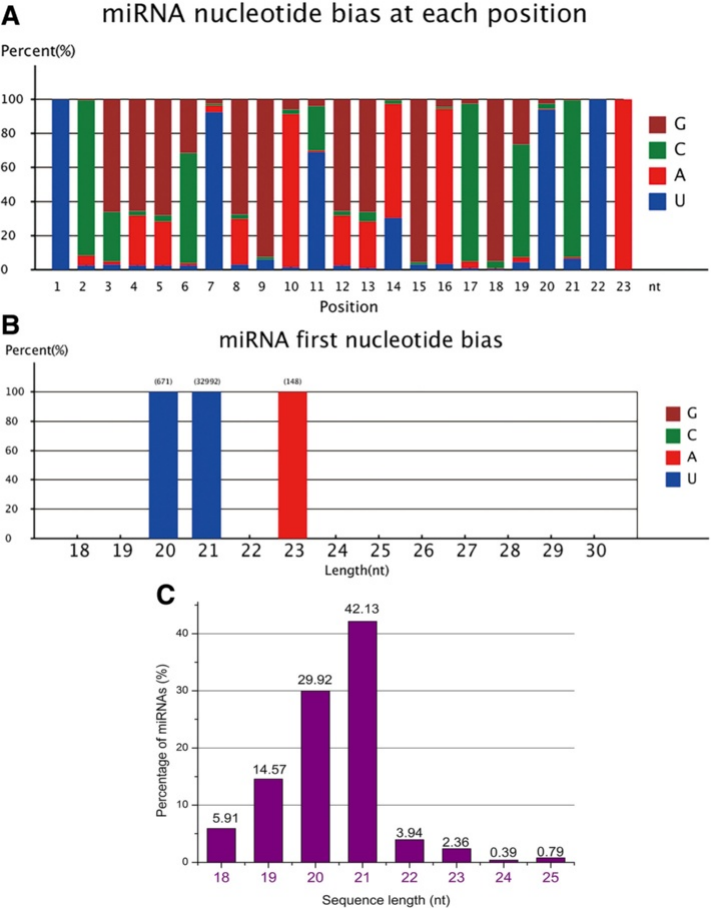
Nucleotides bias of novel miRNAs and size distribution of miRNAs in *P. purpureum.* **a** Percentage of nucleotide bias in novel miRNAs in *P. purpureum.* The novel miRNAs were most biased toward G. **b** Percentage of first-nucleotide bias in novel miRNAs in *P. purpureum.* The novel miRNAs were most biased toward U at the first-base position. **c** Size distribution of miRNAs predicted in *P. purpureum.* As the percentage of miRNAs, the most abundant miRNAs were 19-nt long

The remaining unannotated reads were not sorted into any of the RNA locus categories. The sequences that could be mapped to the *P. purpureum* genomic exon antisense strand, intron and intergenic regions were used to predict novel candidate miRNAs using Mireap software. We screened the *P. purpureum*-specific novel miRNAs based on a series of rigorous screening criteria described in the Methods section. Of these unannotated reads, 5,440,437 (49.86 %) redundant reads and 424,606 (25.79 %) unique reads could be mapped to the genome and were screened to identify 51 novel miRNAs as shown in Additional file 2: Table S1. Figure 2a shows the nucleotide bias at each position and Fig. 2b shows the bias of the first nucleotide in novel *P. purpureum* miRNAs. G was the most biased base (Fig. 2a). The first 5’ nucleotide in the novel miRNAs was biased towards U, in accordance with the character of typical plant miRNA sequences (Fig. 2b) [41].

To identify as many miRNAs as possible in *P. purpureum*, we used rich *P. purpureum* EST assembly data to predict additional potential conserved miRNAs. EST sequences were aligned against the known plant miRNA sequences deposited in miRBase 21.0. Based on the plant miRNA screening criteria described in the Methods section, only nine additional sequences were identified as miRNAs in *P. purpureum* with high confidence (Additional file 2: Table S1). In all, 254 miRNAs, including 203 conserved (194 from RNA-seq and nine from bioinformatics prediction) and 51 novel miRNAs were identified in *P. purpureum* in this study. A portion of the precursor secondary structures can be seen in Additional file 1: Figure S2A–E.

These conserved and novel miRNAs showed variable levels of expression in this study. For example, 1,073,094 additional conserved reads were obtained for miR419b-3p. In contrast, only 6 reads could be obtained for ppu-miR15. Overall, the conserved miRNAs were expressed at higher levels than the novel mRNAs, with an average of 397,431 reads per miRNA. From the distribution of miRNA length in *P. purpureum* (Fig. 2c), we can see that a majority of the sequences are 21 nt in length, in accordance with the standard size of typical plant miRNAs [42].

### Conservation and diversity of predicted miRNA families

**Fig. 3:**
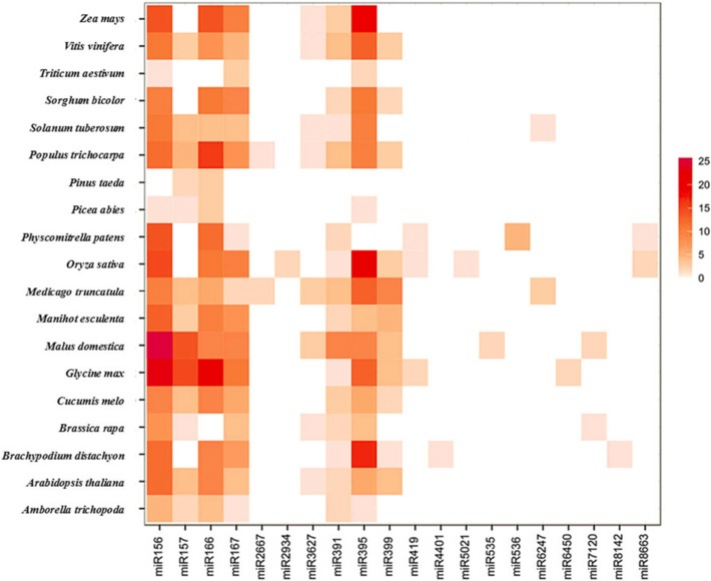
Distribution of conserved miRNA families in different plant species. Data for mature miRNAs in other plant species were from miRBase21.0. Color coding was used to indicate the number of miRNA members, with dark red corresponding to the highest number and white the lowest

To identify potential miRNA families in *P. purpureum*, alignment and cluster searches were performed based on miRNA and precursor sequences. Altogether, 235 potential miRNA families were identified in *P. purpureum* from 254 members (Additional file 3: Table S2). In *P. purpureum*, the largest miRNA families were miR156, miR166 and miR5021, which each had three members. As shown in Table 3, a majority (93.19 %) of the predicted families in *P. purpureum* had only one member. To investigate the conservation and diversity of the predicted miRNA families in different plant species, 20 randomly selected conserved family sequences were aligned against the sequences of 19 plant miRNA families deposited in miRBase 21.0 (Additional file 4: Table S3). As shown in Fig. 3 and Additional file 4: Table S3, the selected families were highly homologous with the 19 plant species investigated, suggesting that potential miRNA-mediated biological regulatory functions may be relatively well-conserved. Ten of the predicted families had homology with *Oryza sativa*, *Medicago truncatula* and *Manihot esculenta*. The miR156 family had homology with all of plant species investigated except for *Pinus taeda*, indicating that this miRNA family might originate from a single sequence in some unknown ancestral species. Based on the distribution of potential miRNA families in the 19 random plant species investigated in this study, which included gymnosperms, angiosperms and bryophyta (Fig. 3), we found that the distribution of these predicted families revealed diversity characteristics in plants even if those families were selected randomly.

**Table 3:** Number of member in each miRNA family identified in *P. purpureum*

Size of family member	Number of miRNA family	Percent of conserved miRNA family (%)	Percent of novel miRNA family (%)
1	219	78.30 %	14.89 %
2	13	2.13 %	3.40 %
3	3	1.28 %	0.00 %

### Validation of *P. purpureum* miRNAs

**Fig. 4:**
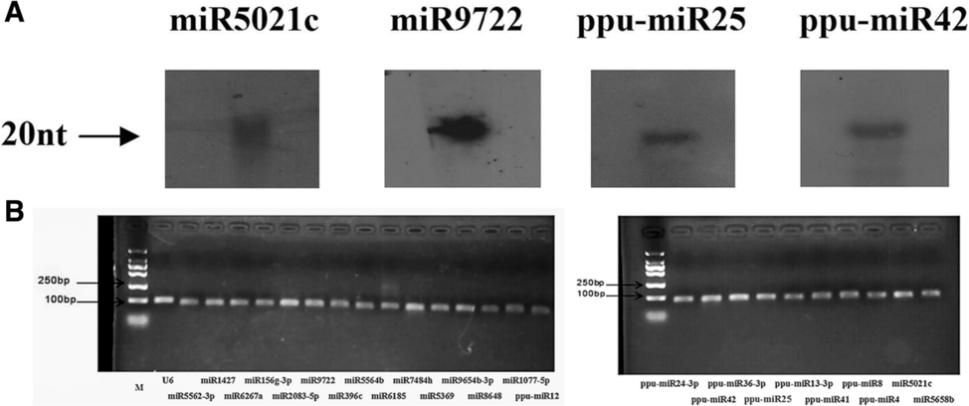
Gel-based detection of *P. purpureum* miRNAs. **a** RNA gel blot hybridization of digoxigenin-labeled probes for four *P. purpureum* miRNAs. **b** Agarose gel of stem-loop qRT-PCR products based on 25 *P. purpureum* miRNAs

To validate the effectiveness and credibility of the high-throughput RNA-seq and bioinformatics analysis, we randomly chose four miRNAs (two conserved and two novel miRNAs) and subjected them to northern blot hybridization, the most direct miRNA validation method [43]. The designed oligonucleotides are described in Additional file 5: Table S4. All four of the miRNAs could be detected by northern blot analysis (Fig. 4a). In addition, 25 miRNAs (nine novel and sixteen conserved miRNAs) were randomly chosen and subjected to stem-loop qRT-PCR detection. The PCR primers used can be seen in Additional file 5: Table S4. As a result of the specificity and sensitivity of stem-loop qRT-PCR [41], all 25 miRNAs could be detected (Fig. 4b). Both detection results had 100 % VRs, suggesting that a majority of the miRNAs identified in *P. purpureum* are effective and credible.

### Target genes of miRNAs in *P. purpureum*

Studies have demonstrated that miRNAs can bind to their targets perfectly or nearly perfectly via complementarity, then post-transcriptionally regulate mRNAs by inducing targeted mRNA cleavage or by repressing translation [44, 45]. Therefore, based on the principle of high miRNA-target complementarity, miRNA targets and target genes can be predicted. To predict as many target genes as possible in *P. purpureum*, both psRobot and TargetFinder were used to predict putative target genes. After removing public targets between miRNAs and their corresponding target genes, 16,339 and 2187 targets were predicted for the conserved and novel miRNAs, respectively (Additional file 1: Figure S3A and B). A total of 14,958 and 2184 target genes from 186 conserved and 41 novel miRNAs was predicted, respectively (Table 4). Altogether, 17,142 target genes were predicted in *P. purpureum*. In addition, 2244 (36.2 %) and 2652 (24.28 %) loci between conserved miRNAs and targets obtained by psRobot and TargetFinder were predicted as potential translational inhibition locations based on psRNATarget analysis (Table 4 and Additional file 6: Table S5). Finally, 428 (51.5 %) and 356 (18.65 %) loci between novel miRNAs and their targets obtained by psRobot and TargetFinder were predicted as potential translational inhibition locations (Table 4 and Additional file 7: Table S6).

**Table 4:** Summary of target genes predicted in *P. purpureum*

	Conserved miRNAs			Novel miRNAs		
Software	miRNA number	Target gene number	Translational inhibition/cleavage loci	miRNA number	Target gene number	Translational inhibition/cleavage loci
psRobot	177	5784	3950/2244	37	792	403/428
TargetFinder	181	12102	8270/2652	41	1661	1553/356
Total	186	14958		41	2184	

### GO enrichment

**Fig. 5:**
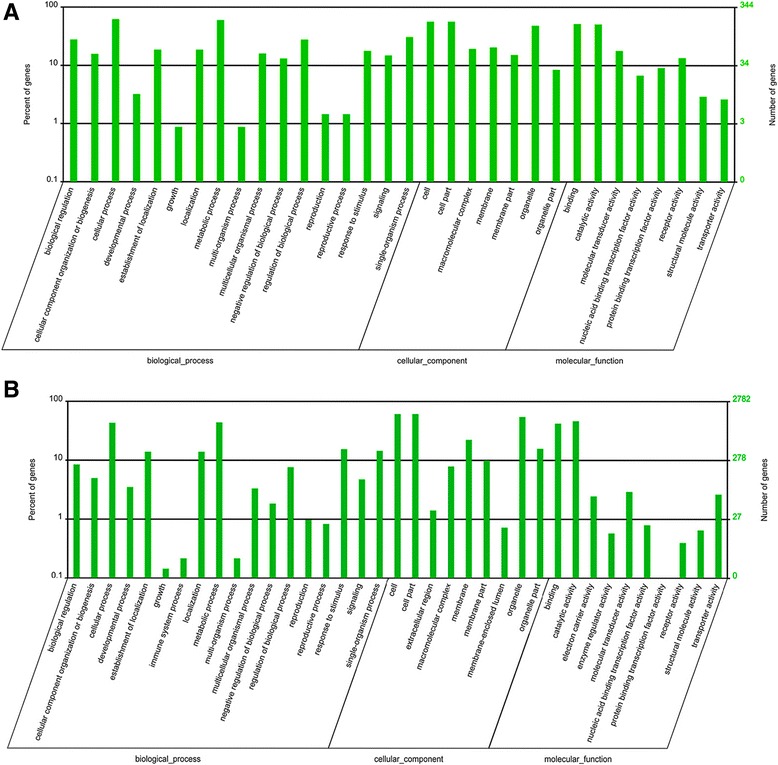
GO classification of target genes. The x-axis shows the diverse biological functions of target genes according to the three GO categories (biological process, cellular component and molecular function). The y-axis shows the percentage and number of these target genes. **a** Figure of GO classification based on the target genes by conserved miRNAs mediated. **b** Figure of GO classification based on the target genes by novel miRNAs mediated

The largest number of target genes regulated by conserved and novel miRNAs was predicted to be in the BP category (Fig. 5a and b). The topological relationships of the ten most enriched GO terms according to their biological process (BP), cellular component (CC) or molecular function (MF) classification are illustrated as directed acyclic graphs (DAGs) in Additional file 1: Figure S4A–C. As indicated by Additional file 8: Table S7 and Additional file 1: Figure S4A–C, the three most significantly enriched GO terms for conserved miRNAs in BP, CC and MF were related to protein import, chloroplast inner membrane and molecular transducer activity, respectively. Similarly, the three most significantly enriched GO terms for novel miRNAs in BP, CC and MF were related to chloroplast relocation, plasma membrane and photoreceptor activity, respectively (Additional file 9: Table S8 and Additional file 1: Figure S5A–C). This study of GO term enrichment may provide some insight into important biological processes in *P. purpureum* such as metabolism regulation [46], growth and development [47], and stress response [48].

### KEGG pathway prediction

**Fig. 6:**
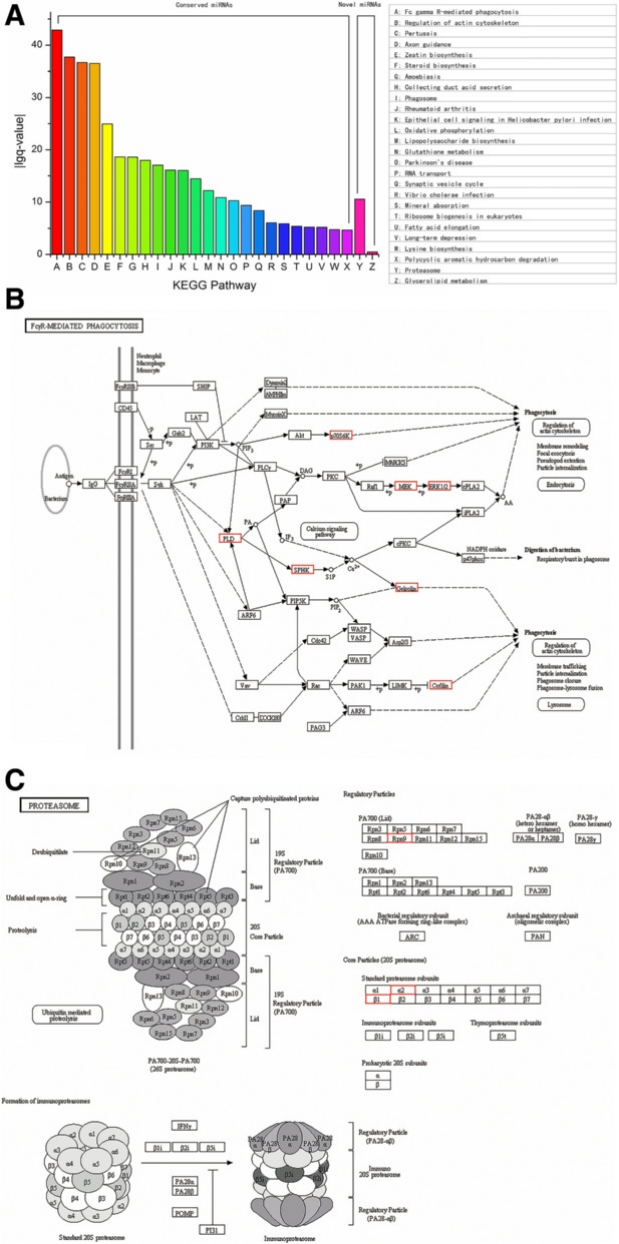
KEGG pathway analysis of target genes. **a** The top 26 significant enriched pathways based on KEGG analysis. The x-axis shows 26 different enriched pathways target gene productions involved in. The y-axis shows the absolute value of lgq. As the pathways with *p*-value < 0.05 and q-value < 0.05, they were predicted as the most significant ones in *P. purpureum.* **b** The most significant enriched pathway for conserved miRNAs. **c** The most significant enriched pathway for novel miRNAs. In the pathway map, small boxes represent proteins or enzymes and red ones indicate the candidate target genes encoding them. The small circles represent metabolites. The arrows represent different metabolic pathways. The detailed introduction about the pathways can be seen online. The website of (B): http://www.genome.jp/kegg-bin/show_pathway?ko04666. The website of (C): http://www.kegg.jp/kegg-bin/show_pathway?mvn03050

Corresponding metabolic pathways can be predicted by KEGG analysis [49]. A pathway map can be used to analyse target gene products and their potential functions during metabolic processes [50]. The most significantly enriched 26 KEGG pathways (24 from conserved miRNAs and two from novel miRNAs) can be seen in Fig. 6a. Based on conserved miRNA regulation in *P. purpureum,* FcγR-mediated phagocytosis was the most significantly enriched pathway (Additional file 10: Table S9, Fig. 6a and b). Similarly, based on novel miRNA regulation, proteasome formation was the most significantly enriched pathway *in P. purpureum* (Additional file 10: Table S9, Fig. 6a and c).

### miRNA-target interaction network

The interaction network between miRNAs, miRNA-target gene and target genes was constructed in Cytoscape 3.0.1 [51]. The completed networks are not shown due to over-dense data for conserved miRNAs and their target genes. However, the two most enriched and cross-linked networks for novel miRNAs and their target genes can be shown clearly. The most highly enriched network for novel miRNAs was ppu-miR40, with 407 relevant target genes (Additional file 1: Figure S6A). The most cross-linked networks for novel miRNAs in this study were ppu-miR30 with 81 target genes, ppu-miR34 with 16 target genes and ppu-miR43 with 22 target genes (Additional file 1: Figure S6B).

## Discussion

As a representative unicellular micro red alga, *P. purpureum* has been a focus of algae researchers around the world [52]. Research hotspots include the functional properties and essential metabolic mechanisms of this alga and the application of its biological active substances. Prior to this study, fundamental information regarding sRNA sequences, especially for miRNAs and their functions, were lacking for this unique red alga, which will be essential for studies above. In this study, we applied high-throughput sequencing combined with bioinformatics methods to identify and characterize miRNAs in *P. purpureum*. Large quantities of sequence information are necessary for sRNA research with this micro-alga, and such sequence information should help address the lack of information for this unique species. Moreover, these research results may serve as a foundation for further exploration and application of the complex metabolic mechanisms of unique bioactive substances found in *P. purpureum*.

Usually, in order to grab the species-specific expressed miRNAs, two or more samples need to process sRNA-seq consisted of one control sample and at least one treatment sample [53]. In our research, the unique pure micro-algae samples with high content of bioactive substances as the introduction said may be hard to obtain under the field conditions [13]. Furthermore, the suitable cultured samples with some specific treatments need a long time to be obtained in the laboratory. As a preliminary qualitative analysis on *P. purpureum* sRNA, only the control samples cultured in the photo bioreactor were performed sRNA deep sequencing in this research. Next, sRNA-seq of the specific treated samples will be completed whose results will be essential to grab the differentially expressed miRNAs of interest in *P. purpureum*.

Completed reference genome should be the primary limiting factor for sRNA-seq. Fortunately, the genome of *P. purpureum* has been published [20]. Regrettably, the published incomplete genome information with the assembly level of contig and deletion of chromosomes in this algae resulted in only 49.86 % reads mapped to the genome successfully (Table 2). However, an incomplete data is better than the inaccurate one [54]. Thus, up to date, the results of sRNA-seq in *P. purpureum* may be more confidence. More and more unmapped reads could be annotated as the more completed genome information published in the future.

Although some methods such as in situ hybridization are powerful tools for miRNA validation, spatial and temporal variation in gene expression remain major limiting factors for miRNA validation in plants. Fortunately, although miRNAs were selected randomly for validation in this study, northern blot and qPCR validation results were positive for all selected miRNAs (Fig. 4a and b). This validation result lends high confidence to the miRNAs identified in this study. Conservation and diversity analysis of miRNA families in different plant species demonstrated that some types of miRNAs are relatively conserved between some species while other types of miRNAs are diverse [55]. Further evolutionary analysis based on comparative genomics in massive and broader plant miRNAs may be necessary to confirm whether this phenomenon is related to species biological evolution [56].

The predicted translational inhibition results for conserved miRNAs (36.2 and 24.28 % based on two target genes prediction programs) indicated that cleavage may be the main mode of post-transcriptional inhibition in *P. purpureum* (Table 4). Many studies have shown that cleavage Ago2 mediated may be the main mode of gene suppression for many known plant miRNAs [57]. Currently, this similar regulation is often called RNA interference [58]. However, for novel miRNAs, two different potential translational inhibition locations (51.5 and 18.65 %) were obtained (Table 4), suggesting that the post-transcriptional regulation process may still be ambiguous for these species-specific miRNAs. Due to the lack of spatial and temporal gene expression analysis, we may underestimate the level of miRNA-mediated translational inhibition. Validation of target genes and expression analysis will be necessary in future studies.

The predicted target genes and the functions of their products may provide valuable clues for research into special biological substances, essential biological processes and metabolisms in *P. purpureum* [59]. For example, according to GO analysis, the target genes enriched in the GO terms for CC (GO: 0042651 for conserved miRNAs and GO: 0044436 for novel miRNAs) (Additional file 8: Table S7 and Additional file 9: Table S8) may regulate the synthesis of the B-phycoerythrin storage location (the thylakoid membrane); these genes are mediated by miR9748, miR5647 and ppu-miR31 [60]. The target genes enriched in the GO terms for MF (GO: 0060229 for conserved miRNAs and GO: 0042578 for novel miRNAs) (Additional file 8: Table S7 and Additional file 9: Table S8) may regulate lipid metabolism and should be closely related to synthesis of PUFA, a unique fatty acid in *P. purpureum*; these genes are regulated by miR1427 and ppu-miR33 [61]. The target genes enriched in the GO terms for BP (GO: 0006950 for conserved miRNAs and GO: 0033554 for novel miRNAs) (Additional file 8: Table S7 and Additional file 9: Table S8) may be related to cellular responses to various exotic stresses and are regulated by a class of miRNAs that includes miR6173, miR8180, and ppu-miR23 [62]. The most interesting target genes in BP (GO: 0035194 for conserved miRNAs) (Additional file 8: Table S7) may be involved in the synthesis of pivotal factors, playing key roles in miR2916- and miR7494e-mediated post-transcriptional gene silencing [63].

Some predicted KEGG metabolic pathways may provide some important assistances for explore the unique micro-algae. Such as the FcγR-mediated phagocytosis (ko04666), the most enrich pathway in which miR9748, miR4994-3p and miR6135k are involved (Additional file 6: Table S5 and Additional file 10: Table S9), is a very interesting metabolic pathway [64]. In its KEGG metabolic map, enriched production of p70 ribosomal S6 kinase (p70S6k), mitogen-activated protein kinase (MEK), and phospholipase D1/2 (PLD) may play important roles in cell phagocytosis, digestion of bacteria and immune regulation in vivo (Fig. 6b) [65]. Further studies will be necessary to confirm whether this phagocytosis pathway is an anti-virus and anti-tumour pathway complementary to EPS in *Porphyridium*. Proteasome formation (ko03050) is another highly enriched pathway in which ppu-miR12 and ppu-miR17 are involved (Additional file 7: Table S6 and Additional file 10: Table S9). Proteasome formation is an essential biological process and plays important roles in cell cycle regulation, cell apoptosis, cellular stress response and immune regulation [66]. The enriched regulatory subunits in the pathway (Rpn9, α2 and β1) are necessary for proteasome formation (Fig. 6c) [67]. Although some studies have demonstrated that *P. purpureum* has anti-virus, anti-tumour, and immunity-enhancing effects in vitro and in vivo [68], the detailed regulatory mechanisms of these effects are still not well understood. The two KEGG pathways mentioned above may provide a starting point for elucidating these molecular mechanisms and will be essential for the development and utilization of this unique red alga in the future.

## Conclusions

A total of 254 miRNAs from 235 miRNA families have been identified in *P. purpureum* by deep sequencing and bioinformatics. Some miRNAs screened randomly in the red algae were validated by northern blot hybridization and stem-loop qRT-PCR. Altogether, 17,142 miRNAs targeted genes and their function were predicted in the unicellular alga by bioinformatics software operation. GO enrichment, KEGG pathway analysis and cytoscape network prediction will provide valuable references for further research on this unique micro-algae.

## Methods

### Algae materials and cultivation

Due to environmental pollution and algae-bacteria symbiosis, it is relatively difficult to obtain highly pure samples of microalgal samples from the wild [69]. To ensure high-quality RNA sequencing (RNA-seq) of *P. purpureum* (No. FACHB-806), we cultured pure algae germplasm obtained from the Institute of Hydrobiology of the Chinese Academy of Sciences (FACHB-collection, Wuhan, China). Cultivation was performed indoors. We conducted the cultivation in a 5.0 L stirred photo bioreactor designed in-house, and cultivation conditions were based on John et al.’s [70] classic artificial seawater medium (ASW) and Chen et al.’s [71] optimized photo bioreactor parameters. The medium consisted of 2.8 g/L NaHCO_3_, 3.0 g/L NaNO_3_, 0.03 g/L KH_2_PO_4_, 0.9 mg/L V_B1_, 2.7 μg/L V_B12_ and 0.11 mg/L Fe-ethylene diamine tetraacetic acid (EDTA) mixed with sterile seawater and distilled water (*v*:*v* = 1:1) at pH = 7.6. The experimental photo flux density was 1000 μmolm^−2^s^−1^, and the temperature was 25 °C. The culture was agitated at 200 rpm and aerated at 0.71 vvm. We collected microalgal tissues when stationary phase was reached after approximately 6–8 days. Samples were immediately frozen in liquid nitrogen and stored at −80 °C.

### sRNA library construction and deep sequencing

An sRNA library was constructed and deep sequencing was performed based the methods reported by Hafner et al. [72] and Wei et al. [73]. Total RNA was isolated from enriched tissues with TRIzol reagent (Invitrogen, Carlsbad, California, USA). Only RNA samples with high purity (OD260/280 between 1.8 and 2.2) and high integrity (RNA integrity number of 7.0 or higher) were used to construct the sRNA library [74]. In this study, a high quality sRNA library was constructed using approximately 10 μg of sRNA that was isolated using an Illumina TruSeq Small RNA Sample Prep kit (Illumina, San Diego, California, USA). Then, deep sequencing was performed on an Illumina HiSeqTM 4000 (Illumina) according to the manufacturer’s protocol. Quality assessment of the sRNA library was performed on an Agilent 2100 Bioanalyzer (Agilent, Palo Alto, California, USA) and StepOnePlus Real-Time PCR System (ABI, Carlsbad, California, USA). The raw data of sRNA-seq have been submitted to the NCBI Sequence Read Archive database.

### Bioinformatics analysis of sRNA

Only clean reads remained after removal of contaminating (5’ adaptor contaminants, no insert tags, oversize insertions, poly (A) tags and small tags) and low-quality reads. Reads with the same sequences were named unique reads. The numbers and lengths of clean reads were calculated. Clean reads were then annotated according to the following steps [75]: (i) clean reads were mapped to the *P. purpureum* genome announced in June 2013 using SOAP (http://soap.genomics.org.cn/) [76], (ii) mapped reads were aligned to *P. purpureum* ESTs in GenBank (July, 2014) using the basic local alignment search tool (BLAST), (iii) aligned reads were then aligned to precursor/mature plant miRNAs in miRBase 21.0 (July, 2014) using BLASTn analysis, allowing for two mismatches and three gaps, (iv) precursors of reads meeting the above requirements were predicted as known miRNAs precursors, and their potential stem-loop structures was determined using Mireap (http://sourceforge.net/projects/mireap/), (v) in addition to konwn miRNAs, other sRNA reads such as tRNAs, rRNAs, snRNAs, snoRNAs annotated in Rfam 11.0 (http://rfam.sanger.ac.uk) [77] and GenBank were obtained via alignment analysis, and any remaining unannotated sRNAs were stored for further prediction of novel miRNAs.

### Identification of conserved miRNAs

The potentially known miRNAs predicted above will be a subset of the conserved miRNAs in *P. purpureum*. To obtain as many miRNAs as possible in *P. purpureum*, we identified additional candidate conserved miRNAs by aligning the 386,903 ESTs of *P. purpureum* in Genbank (December, 2014) against 6992 plant miRNAs and 8450 precursors in miRBase 21.0 (December, 2014) using the methods described by Zhang et al. [78]. The following screening criteria were used to identify potential conserved miRNAs: the sum of mismatches and gaps should be no more than three for both the mature miRNA and its precursor; the length of the mature miRNA should be 18–25 nt; the candidate RNA should not have been previously identified as an rRNA, tRNA snRNA, snoRNA or other non-miRNA; and the precursor should have a perfect stem-loop hairpin structure. The additional conserved miRNAs identified in this manner as well as known miRNAs were considered conserved miRNAs in this study.

### Identification of novel miRNAs

The clean reads that mapped to the *P. purpureum* genome but could not be assigned to one of the aforementioned RNA classes were used to predict novel miRNA candidates in *P. purpureum*. Novel miRNAs and their precursors were identified by aligning unclassed clean reads against the ESTs of *P. purpureum* in Genbank (March, 2015) with Mireap software (http://sourceforge.net/projects/mireap/) [79]. After filtering out unreasonable reads, which included reads without perfect hairpin structures, reads with 5’ and 3’ overhangs greater than 2 nt in length, reads with loops and bulges greater than 4 nt in length on either strand and reads with minimum free energies (MFE) less than −18 kcal/mol [80], the remaining high-confidence reads were considered potential novel *P. purpureum-*specific miRNAs.

### Prediction of miRNA family

After aligning miRNAs and their precursors against plant miRNAs deposited in miRBase using Clustal X 2.0 and MEGA 5.0 [81, 82], only sequences with greater than 98 % homology and fewer than two mismatches were predicted to be potential miRNA family members from the conserved miRNA families in plants. Novel miRNA genes could be predicted as species-specific miRNA families [83]. BLASTn comparisons were performed between twenty random conserved miRNA families in *P. purpureum* and 19 random plant miRNA families stored in miRBase 21.0 (June, 2015); results from these comparisons may indicate the conservation and diversity of these identified miRNA families in different plant species.

### Northern blot validation

Two conserved miRNAs (miR5021c and miR9722) and two novel miRNAs (ppu-miR25 and ppu-miR42) were selected randomly and subjected to northern blot analysis [84]. According to Jin et al.’ methods [85], northern blots can be conducted based on hybridization with miRNA-complementary DNA oligonucleotides labelled with digoxigenin (DIG) (Roche, Basel, Switzerland). The oligo sequence synthesis was completed by Takara Bio. Inc. (Tokyo, Japan). After the incubation and wash steps, final RNA bands on membranes can be photographed with X-ray exposure.

### Stem-loop qRT-PCR validation

Twenty-five miRNAs (16 conserved miRNAs and nine novel miRNAs) were randomly selected for stem-loop qRT-PCR. Stem-loop qRT-PCR was performed according to the methods described by Varkonyi-Gasic et al. [86] to further verify our identification results. The primers sequences synthesis were completed by Geneseed Biotech Co., Ltd (Guangzhou, China). Approximately 1.0 μg of total RNA was used to synthesize cDNA with a HiScript 1st Strand cDNA Synthesis Kit (Vazyme Biotech, New York, USA). qPCR was carried out with ChamQ SYBR qPCR Master Mix (Vazyme Biotech) and a BIO-RAD IQ5 real-time PCR system (BIO-RAD, Hercules, California, USA). Three replicates were performed for all samples in this study.

### Prediction of target genes

The putative genes targeted by the miRNAs identified in *P. purpureum* were identified using two different plant-targeted gene prediction software packages: psRobot (http://omicslab.genetics.ac.cn/psRobot/) [87] and TargetFinder (http://carringtonlab.org/) [88]. Identified miRNA sequences were used as queries against *Porphyridium* transcripts and ESTs deposited in Genbank (July, 2015). Under the premises of perfect complementary and high homology between miRNAs and their targets in plants, potential targets were obtained with the following software settings [89]: (i) zero deletions and insertions; (ii) fewer than three mismatches in all, with fewer than one mismatch in positions 1–9 and zero mismatches at positions 10 and 11; (iii) a perfect duplex in positions 8–12; (iv) zero nucleotides contained within loops or bulges in either strand; (v) 5’ and 3’ overhangs no more than two nucleotides in length, and (vi) MFE values between miRNAs and complementary sequences less than −18 kcal/mol. Using the method reported by Dai et al. [90], psRNATarget (http://plantgrn.noble.org/psRNATarget/) was used to predict potential translational inhibition type based on whether one mismatch was detected in the central complementary region of the miRNA (9–11 nt).

### Functional annotation of target genes

To determine the function of target genes, GO and KEGG enrichment were conducted. GO term annotation was assigned to target genes using Blast2GO 3.1 analysis [91]. The enrichment analysis of GO terms was conducted by running the GO Enrichment Analysis Software Toolkit [92]. Only terms with corrected *p* values < 0.05 were defined as significantly enriched terms in the target gene candidates. The AmiGO tool from the Gene Ontology Consortium (http://geneotology.org/) was used to classify target genes according to GO-controlled vocabularies, describing gene products in terms of BP, CC and MF [93]. Using Goseq package analysis, the DAG of the ten most enriched terms was generated based on the three ontologies [94]. Similarly, target genes were assigned KEGG terms and subjected to enrichment analysis. Based on the correlation between target genes and their putative functional products as determined by KOBAS 2.0 (http://kobas.cbi.pku.edu.cn/home.do) [95], the corresponding metabolic pathways of the enriched target genes were predicted with a class of pathway IDs [96]. Significantly enriched KEGG pathways with *p*-values < 0.05 and *q*-values < 0.05 were the focus of our research [97]. A potential pathway map was obtained by running KegSketch (http://genome.jp/kegg/) [98].

### Construction of miRNA-target interaction network

Based on the target genes and their corresponding miRNAs, an miRNA-target interaction network was constructed and was visualized in Cytoscape 3.0.1 (http://www.cytoscape.org) [99]. This network revealed the potentially complicated correlation between diverse miRNAs, miRNA-target genes and target genes.

## Abbreviations

ASW, artificial seawater medium; BLAST, basic local alignment search tool; BP, biological process; CC, cellular component; CVB3, coxsackie virus B3; DAGs, directed acyclic graphs; DIG, digoxigenin; EDTA, ethylene diamine tetraacetic acid; EPS, exo-polysaccharides; ESTs, expressed sequence tags; GO, gene ontology; Hep-2, human epidermoid cancer cells; HSV, herpes simplex virus; IL-2, interleukin-2; KEGG, Kyoto Encyclopedia of Genes and Genomes; MEK, mitogen-activated protein kinase; MF, molecular function; MFE, minimum free energies; miRNA, microRNA; NK, natural killer cell; p70S6k, p70 ribosomal S6 kinase; PLD, phospholipase D1/2; PUFA, polyunsaturated fatty; qRT-PCR, quantitative real time RT-PCR; RSV, respiratory syncytial virus; SMMC7721, spontaneous monocyte-mediated cytotoxity 7721; sRNA, small RNA; sRNA-seq, small RNA deep sequencing; VR, validation rate; VZV, varicella zoster virus

## Additional files

Additional file 1: Figures S1-S6. An introduction has been included within the Additional file itself. (PDF 29955 kb) Additional file 2: Table S1. Predicted conserved and novel miRNAs in *P. purpureum*. (XLS 3244 kb) Additional file 3: Table S2. Predicted miRNA families in *P. purpureum*. (XLS 51 kb) Additional file 4: Table S3. Conserved miRNAs distributions in different plant species. (XLS 155 kb) Additional file 5: Table S4. The oligo-nucleotide sequences and primer sequences used for northern blot and stem-loop qRT-PCR. (XLS 24 kb) Additional file 6: Table S5. Targets prediction of conserved miRNAs based on psRobot and TargetF inder analysis. (XLS 4535 kb) Additional file 7: Table S6. Targets prediction of novel miRNAs based on psRobot and TargetFinder analysis. (XLS 753 kb) Additional file 8: Table S7. GO analysis of conserved miRNAs in *P. purpureum*. (XLS 684 kb) Additional file 9: Table S8. GO analysis of novel miRNAs in *P. purpureum*. (XLS 283 kb) Additional file 10: Table S9. KEGG pathway analysis *in P. purpureum*. (XLS 421 kb)
