# Supplementary material for: Identification of conserved and novel microRNAs in Porphyridium purpureum via deep sequencing and bioinformatics
Source: BMC Genomics. 2016 Aug 11;17:612. doi: 10.1186/s12864-016-2985-7 (PMC4981961; doi:10.1186/s12864-016-2985-7)
Supplement: Additional file 1: Figures S1-S6. — An introduction has been included within the Additional file itself. (PDF 29955 kb) [file 12864_2016_2985_MOESM1_ESM.pdf]

BMC Genomics

Identification of conserved and novel microRNAs in *Porphyridium purpureum* via deep sequencing and bioinformatics

Fan Gao, Fangru Nan, Jia Feng, Junping Lv, Qi Liu, Shulian Xie*

School of Life Science, Shanxi University, Taiyuan, 030006, P. R. China.

*To whom correspondence should be addressed. E-mail: xiesl@sxu.edu.cn

Present Address: School of Life Science, Shanxi University, Taiyuan, 030006, P. R. China

Supplementary Figure Legends

Fig. S1. Pie chart of different sRNAs distribution in *P. purpureums*

(A) Frequency distribution of total sRNA reads in *P. purpureum*. (B) Frequency distribution of unique sRNA reads in *P. purpureum*.

Fig. S2. Secondary structure of predicted pre-miRNAs in *P. purpureum*

(A) Stem-loop structure of the precursor of miR156g-3p. (B) Stem-loop structure of the precursor of miR5021c. (C) Stem-loop structure of the precursor of miR5658b. (D) Stem-loop structure of the precursor of ppu-miR34-3p. (E) Stem-loop structure of the precursor of ppu-miR40.

Fig. S3. Venn diagram of targets predicted based on psRobot and TargetFinder in *P. purpureum*

(A) Venn diagram of targets with conserved miRNAs. (B) Venn diagram of targets with novel miRNAs. The violaceous part stands for the number of targets predicted by TargetFinder. The green part stands for the number of targets predicted by psRobot. The public part is the number of targets shared by the two software above.

Fig. S4. DAG of the top 10 most enriched GO terms with conserved miRNAs

The DAG shows the topological relationships of the 10 most enriched GO terms. Circles and boxes with different colors represent enriched GO terms, in biological process, cellular component and molecular function categories. (A) DAG of terms enriched with conserved miRNAs in the biological process category. (B) DAG of terms enriched with conserved miRNAs for cellular components. (C) DAG of terms enriched with conserved miRNAs for molecular function.

Fig. S5. DAG of the top 10 most enriched GO terms with novel miRNAs

(A) DAG of terms enriched with novel miRNAs in the biological process category. (B) DAG of terms enriched with novel miRNAs for cellular components. (C) DAG of terms enriched with novel miRNAs for molecular function.

Fig. S6. Subset of the Cytoscape networks constructed in *P. purpureum*

The Cytoscape network reflects the relationships among novel miRNAs, novel miRNAs-target genes and target genes in *P. purpureum*. In the networks, node stands for target genes or miRNAs, and edge reflects interaction among various nodes. (A) The most enriched network predicted in *P. purpureum*. (B) The most cross-linked network predicted in *P. purpureum*.

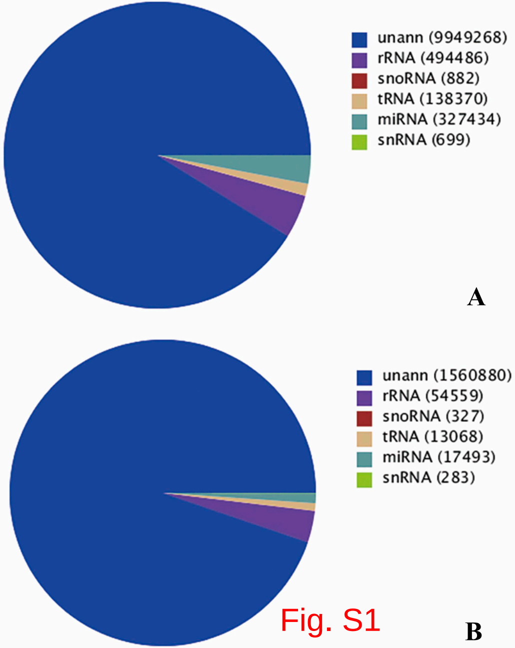
unann (9949268)
rRNA (494486)
snoRNA (882)
tRNA (138370)
miRNA (327434)
snRNA (699)
A
unann (1560880)
rRNA (54559)
snoRNA (327)
tRNA (13068)
miRNA (17493)
snRNA (283)
B

Fig. S1

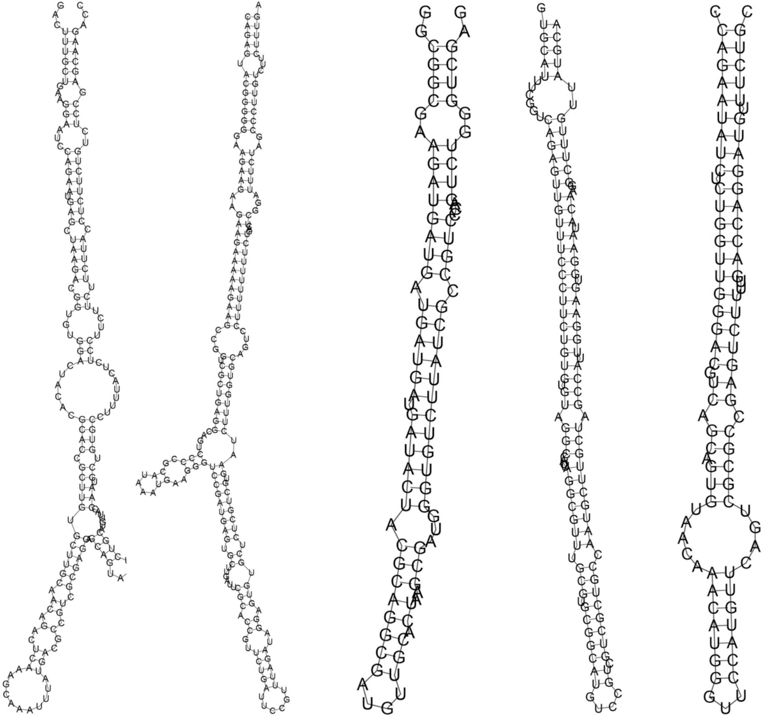

A B C **Fig. S2** D E

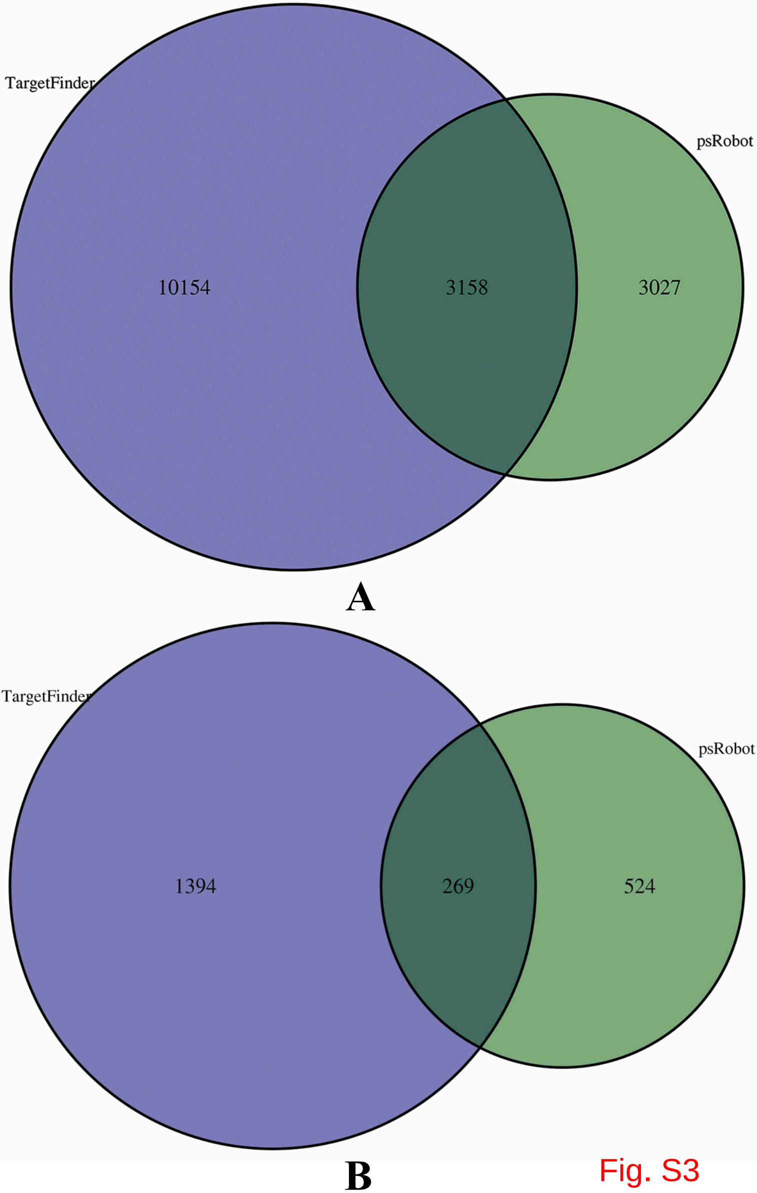
TargetFinder
psRobot
10154
3158
3027
A
TargetFinder
psRobot
1394
269
524
B

Fig. S3

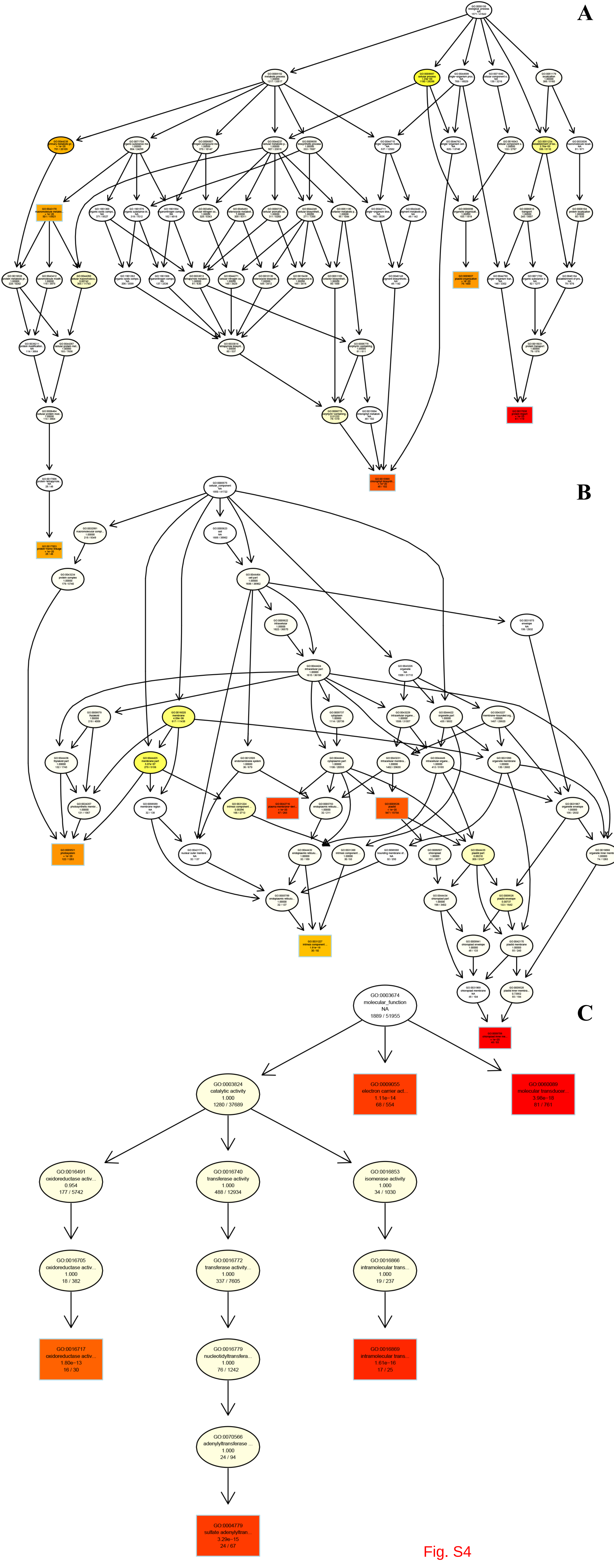
A
B
C
GO:0003674
molecular_function
NA
1889 / 51955
GO:0003824
catalytic activity
1.000
1280 / 37689
GO:0009055
electron carrier act...
1.11e-14
68 / 554
GO:0060089
molecular transducer...
3.98e-18
81 / 761
GO:0016491
oxidoreductase activ...
0.954
177 / 5742
GO:0016740
transferase activity
1.000
488 / 12934
GO:0016853
isomerase activity
1.000
34 / 1030
GO:0016705
oxidoreductase activ...
1.000
18 / 382
GO:0016772
transferase activity...
1.000
337 / 7605
GO:0016866
intramolecular trans...
1.000
19 / 237
GO:0016717
oxidoreductase activ...
1.80e-13
16 / 30
GO:0016779
nucleotidyltransfera...
1.000
76 / 1242
GO:0016869
intramolecular trans...
1.61e-16
17 / 25
GO:0070566
adenylyltransferase ...
1.000
24 / 94
GO:0004779
sulfate adenylyltran...
3.29e-15
24 / 67

Fig. S4

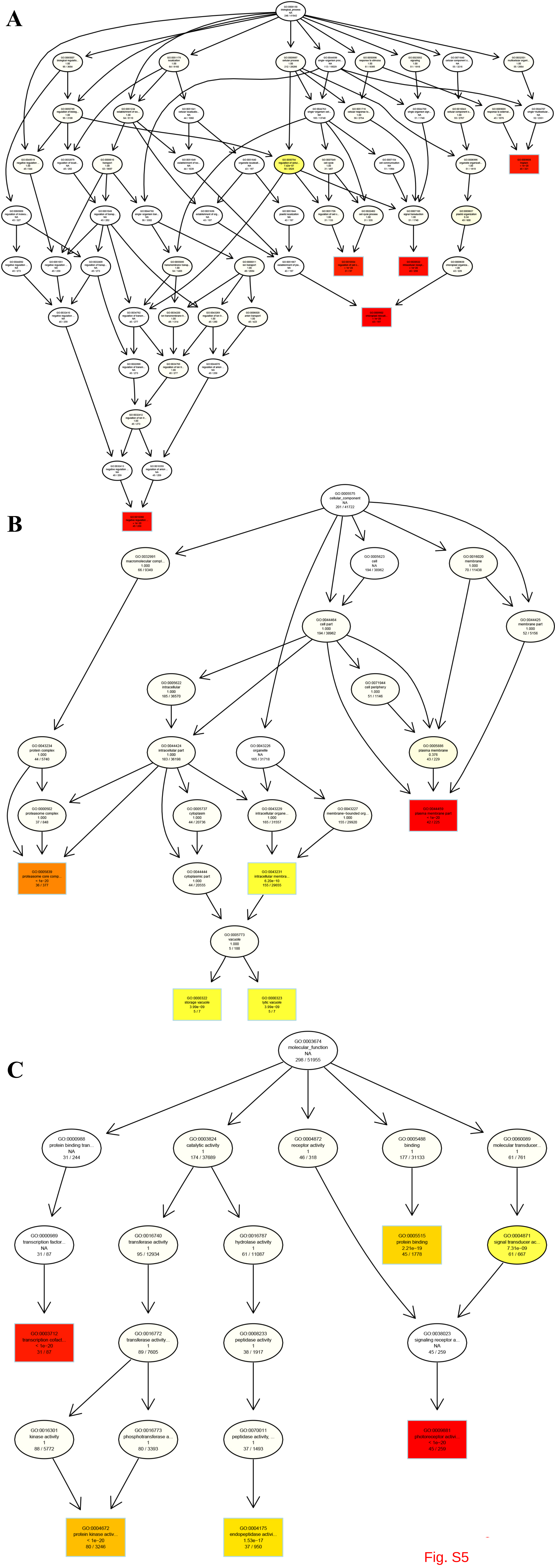
A
B
GO:0005575
cellular_component
NA
201 / 41722
GO:0032991
macromolecular compl...
1.000
66 / 9349
GO:0005623
cell
NA
194 / 38962
GO:0016020
membrane
1.000
70 / 11438
GO:0044464
cell part
1.000
194 / 38962
GO:0044425
membrane part
1.000
52 / 5156
GO:0005622
intracellular
1.000
185 / 36570
GO:0071944
cell periphery
1.000
51 / 1146
GO:0043234
protein complex
1.000
44 / 5740
GO:0044424
intracellular part
1.000
183 / 36198
GO:0043226
organelle
NA
165 / 31718
GO:0005886
plasma membrane
0.370
43 / 229
GO:0000502
proteasome complex
1.000
37 / 848
GO:0005737
cytoplasm
1.000
44 / 20736
GO:0043229
intracellular organe...
1.000
165 / 31557
GO:0043227
membrane-bounded org...
1.000
155 / 29920
GO:0044459
plasma membrane part
< 1e-20
42 / 225
GO:0005839
proteasome core comp...
< 1e-20
36 / 377
GO:0044444
cytoplasmic part
1.000
44 / 20555
GO:0043231
intracellular membra...
6.20e-10
155 / 29655
GO:0005773
vacuole
1.000
5 / 188
GO:0000322
storage vacuole
3.99e-09
5 / 7
GO:0000323
lytic vacuole
3.99e-09
5 / 7
C
GO:0003674
molecular_function
NA
298 / 51955
GO:0000988
protein binding tran...
NA
31 / 244
GO:0003824
catalytic activity
1
174 / 37689
GO:0004872
receptor activity
1
46 / 318
GO:0005488
binding
1
177 / 31133
GO:0060089
molecular transducer...
1
61 / 761
GO:0000989
transcription factor...
NA
31 / 87
GO:0016740
transferase activity
1
95 / 12934
GO:0016787
hydrolase activity
1
61 / 11087
GO:0005515
protein binding
2.21e-19
45 / 1778
GO:0004871
signal transducer ac...
7.31e-09
61 / 667
GO:0003712
transcription cofact...
< 1e-20
31 / 87
GO:0016772
transferase activity...
1
89 / 7605
GO:0008233
peptidase activity
1
38 / 1917
GO:0038023
signaling receptor a...
NA
45 / 259
GO:0016301
kinase activity
1
88 / 5772
GO:0016773
phosphotransferase a...
1
80 / 3393
GO:0070011
peptidase activity, ...
1
37 / 1493
GO:0009881
photoreceptor activi...
< 1e-20
45 / 259
GO:0004672
protein kinase activ...
< 1e-20
80 / 3246
GO:0004175
endopeptidase activi...
1.53e-17
37 / 950

Fig. S5

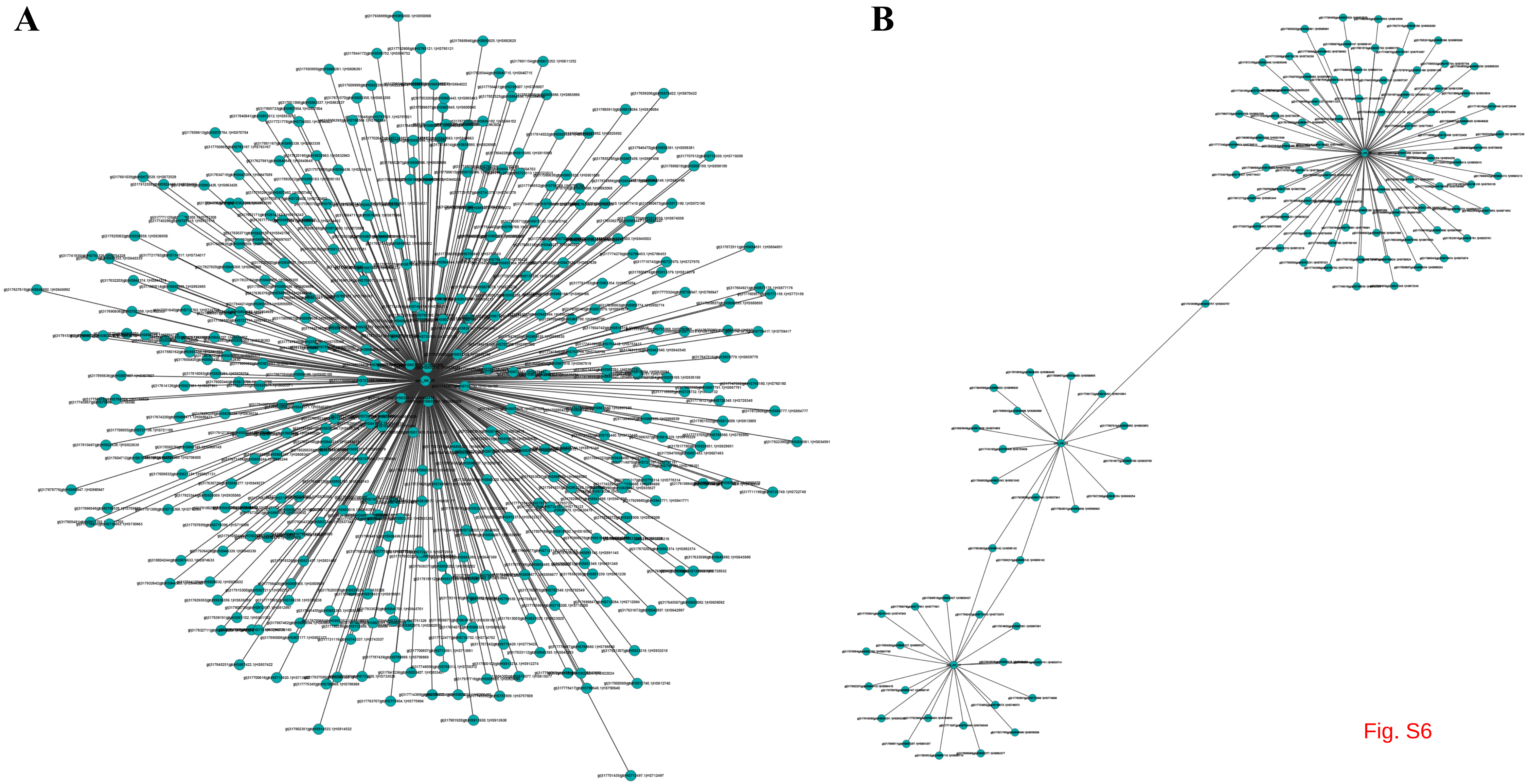

Fig. S6
